# Neighborhood walkability and cardiometabolic disease in Texas

**DOI:** 10.1038/s41598-025-94192-x

**Published:** 2025-03-19

**Authors:** Mei Yang, Tiankai Wang

**Affiliations:** Department of Health Informatics and Information Management, Texas State University, Round Rock, TX USA

**Keywords:** Neighborhood walkability, Cardiometabolic diseases, BMI, Diabetes, Diseases, Risk factors, Environmental impact

## Abstract

Cardiometabolic diseases (CMDs) affect significant numbers of adults in the United States, with 11% diagnosed with diabetes and 10% with cardiovascular diseases. Walking plays a crucial role in reducing health risks, particularly obesity and diabetes. We aim to explore the association between neighborhood walkability and CMD measures in Texas, while controlling for age, sex, racial/ethnic background, and family history of diabetes. We collected 1994 observations for the year 2019, merging data from the Texas Behavioral Risk Factor Surveillance System and the Environmental Protection Agency. We employed multilevel linear regression and multilevel logistic regression analyses to assess the association between CMD measures and neighborhood walkability. Our findings revealed that higher neighborhood walkability is significantly associated with a lower body mass index (BMI) (β = − 0.28, CI − 0.45 to − 0.10) and a reduced risk of diabetes (OR 0.93, CI 0.86–0.99), indicating that when walkability increases by one unit, an individual’s BMI decreases by 0.28 kg/m^2^ and the odds of having diabetes decrease by 7%. We also found that African Americans living in communities with lower walkability scores compared to other racial/ethnic groups. Our findings highlight the need for urban planning policies promoting walkable neighborhoods, suggesting community-based approaches to health promotion.

## Introduction

Cardiometabolic diseases (CMDs), a group of common chronic diseases that affect the cardiovascular system (heart, blood, and blood vessels) and metabolic health, remain the number one cause of mortality worldwide^1,2^. Approximately 17.9 million people globally die from cardiovascular diseases (CVDs) every year^2^. In the United States, CMDs affect a significant number of adults, with approximately 11% diagnosed with diabetes and 10% with CVDs^3^. Although these diseases are prevalent, they are often preventable by controlling the main risk factors, such as an unhealthy diet and physical inactivity.

Neighborhood conditions, such as walkability, are increasingly recognized as important for cardiometabolic health. Walkability is defined as a measure of whether the built environment of a neighborhood encourages people to walk^4^. A walkable neighborhood can promote physical activity, motivating people to walk for recreation and transportation^5^. U.S. adults living in more walkable areas report more walking for both transportation and leisure, especially in urban areas^6^. Additionally, walking can benefit people’s physical and mental health by reducing rates of obesity, diabetes^7^, and other chronic diseases^8^. Adults who perceive higher benefits from exercise and face fewer barriers to exercise are more likely to participate in physical activity. Consequently, adults with higher physical activity levels have lower cardiometabolic risks^9^. This highlights the importance of creating a walkable environment to promote physical activity and reduce cardiometabolic risks.

Walk Score is a widely used method to assess neighborhood walkability. It focuses on proximity to amenities like restaurants, parks, and stores but overlooks critical factors such as pedestrian infrastructure, safety concerns, and the needs of specific populations^10^. This can result in misleading scores, particularly in areas with close amenities but poor pedestrian environments^11^. Urban areas tend to score higher due to a high density of amenities, while suburban and rural areas, which may have good infrastructure but fewer amenities, often score lower^12^. In contrast, the National Walkability Index includes a broader range of factors, such as street intersection density (pedestrian-oriented intersections), proximity to transit stops, and diversity of land uses, offering a more holistic measure of walkability^13^. It also uses nationally standardized data, making it valuable for consistent regional comparisons, as well as for national policy-making and urban planning^13^. In summary, while Walk Score provides a simple and accessible measure of walkability based on proximity to amenities, the National Walkability Index offers a more comprehensive and infrastructure-focused assessment, better suited for detailed research and policy development.

Previous studies found that environmental, social, and behavioral factors are associated with cardiometabolic health^14–20^. The built environment, such as neighborhood walkability, is an important factor influencing cardiometabolic health^14–16^. Walkability is an exposure, while cardiometabolic measures are the outcomes, with walking as a potential pathway in this relationship. For example, walking partially mediated the association between perceived environmental features and metabolic syndrome^14^. Certain cardiometabolic risks are directly associated with neighborhood walkability, such as obesity, type 2 diabetes, systolic blood pressure, and hypertension^15,16^. From social and behavioral perspectives, studies have identified various factors associated with cardiometabolic outcomes. For example, individual factors such as age, male sex, household status, low education, alcohol consumption, and smoking, as well as neighborhood-level socioeconomic status, have been linked to cardiometabolic health^17,18^. Furthermore, unhealthy lifestyles, such as physical inactivity, smoking, and unhealthy diet, increase the risk of CMDs^19,20^. These studies highlight that social, behavioral, and environmental factors collectively influence cardiometabolic health. However, most studies have failed to explore the influence of racial/ethnic background on CMD outcomes.

Some studies have explored neighborhood walkability in relation to different CMD measures across various locations in the United States. For instance, Braun et al. examined the association between walkability and cardiometabolic risk across six regions: Forsyth County, North Carolina; New York, New York; Baltimore, Maryland; St. Paul, Minnesota; Chicago, Illinois; and Los Angeles, California^21^. However, this study used biomarkers such as glucose, triglycerides, high-density lipoprotein and low-density lipoprotein cholesterol, systolic and diastolic blood pressure, and waist circumference as CMD measures, and Walk Score as the measure of walkability. Other studies have investigated the relationship between walkability and specific CMD indicators such as BMI^22^, obesity^23^, and cardiovascular risk^24^ separately across the United States. However, these studies typically focused on a single CMD measure, and the methods for assessing walkability varied.

Our study aims to address these gaps by incorporating a more diverse and comprehensive list of direct CMD outcome measures and using the National Walkability Index to assess neighborhood walkability. The main objective of this study is to explore the association between neighborhood walkability and the incidence of CMDs in Texas, while adjusting for factors such as age, sex, family history of diabetes, and racial/ethnic background. Our hypothesis is that walkability is negatively associated with CMD outcomes. Another objective is to assess the relationship between walkability and different racial/ethnic groups. We hypothesize that African American individuals live in communities with lower walkability scores. We selected Texas as the geographic setting for this study in response to the pressing need for improved CMD outcomes in Texas. Texas has poorer cardiometabolic health outcomes, with CVDs being one of the leading causes of death among Texans^25^. There is an urgent need to examine the associated risk factors and promote strategies to reduce morbidity and mortality in Texas. As the second largest and most populous state in the U.S., Texan cities vary widely in environmental and demographic factors. For example, health disparities are more significant in some Texan cities, such as El Paso^26^. Furthermore, Texas has experienced significant population growth^27^, leading to the development of more new residential communities. The need to build these new communities makes our research particularly meaningful in urban planning. In summary, Texas serves as a relevant and unique area of study due to its healthcare needs, diverse cities, and rapid urbanization.

## Materials and methods

### Data source

We obtained cardiometabolic data at the individual level from the Texas Behavioral Risk Factor Surveillance System (BRFSS). The CMDs measures include body mass index (BMI), high blood pressure, diabetes, heart disease, CVDs, and physical activity. The unit of BMI measurement is kg/m^2^. Physical activity was measured using physical activity index, and it was categorized as either met aerobic recommendations or not. High blood pressure, diabetes, heart disease, and CVDs are binary data: either diagnosed with the disease or not. The BRFSS data also includes other individual variables such as age, sex, family history of diabetes, and racial/ethnic background. Family history of diabetes was categorized as Yes and No. The racial/ethnic background was categorized into four groups: White, African American, Hispanic, and Other or Multiracial. Here, Multiracial means participants with more than one racial background; Other race means participants with a race other than White, African American, and Hispanic.

We obtained the National Walkability Index from the Environmental Protection Agency (EPA). The National Walkability Index was measured based on the built environment: street intersection density, proximity to transit stops, and diversity of land uses^13,28^. It scales from 1 to 20. The higher the value, the higher the walkability. It was collected at the block group level, a unit of census geography that is smaller than a census tract and larger than a census block. We merged the National Walkability Index into the zip code level in order to merge with the cardiometabolic data. In other words, each individuals’ walkability was measured using the National Walkability Index at the zip code level, which is the smallest geographic area information that cardiometabolic data contains.

### Statistical and visual analysis

Because this study aggregated the National Walkability Index at the zip code level to measure neighborhood walkability for each individual, while other variables are at the individual level, we adopted multilevel regression analyses. These analyses explored the association between walkability and multiple CMD measures, including BMI, high blood pressure incidents, diabetes incidents, heart disease incidents, CVDs, and physical activity. We also controlled other variables in the analysis, including age, sex, family history of diabetes, and racial/ethnic background. We employed a multilevel linear regression when analyzing BMI and used multilevel logistic regressions when analyzing other binary CMDs measures.

We first calculated the characteristics of the walkability index for each racial/ethnic category. To better understand the relationship between walkability and different racial/ethnic groups, we performed a univariate linear regression analysis with walkability and racial/ethnic groups. We further analyzed the correlation between walkability and the African American population to examine whether they live in communities with lower walkability scores. We then conducted a pairwise correlation analysis using the pwcorr function in Stata 17.0 to explore the correlation between walkability and African American population. Additionally, we used ArcGIS Pro to create a thematic map to visually present the correlation between walkability and the African American population.

## Results

The final dataset contains 1994 observations for the year 2019. The National Walkability Index scores from 5.05 to 13.89 (Table 1). Compared to other racial/ethnic groups, African American group had the lowest average walkability (mean = 7.04), while the Hispanic group had the highest average walkability (mean = 8.62).

**Table 1:** Characteristics of each variable (N = 1994).

Variables	Value range/number of participants (%)	Mean	Standard deviation	Minimum	Maximum
Body mass index (BMI)	13.6–77.76	28.85	6.84	–	–
High blood pressure					
No	1018 (51.28)	–	–	–	–
Yes	967 (48.72)	–	–	–	–
Diabetes					
No	1606 (80.74)	–	–	–	–
Yes	383 (19.26)	–	–	–	–
Heart disease					
No	1766 (90.29)	–	–	–	–
Yes	190 (9.71)	–	–	–	–
Cardiovascular diseases (CVDs)					
No	1691 (86.36)	–	–	–	–
Yes	267 (13.64)	–	–	–	–
Physical activity index					
Not met aerobic recommendations	881 (49.75)	–	–	–	–
Met aerobic recommendations	890 (50.25)	–	–	–	–
National walkability index	5.05–13.89	8.00	1.96	–	–
Age	18–99	58.51	18.38	–	–
Family history of diabetes					
No	1090 (54.66)	–	–	–	–
Yes	904 (45.34)	–	–	–	–
Sex					
Female	1189 (59.63)	–	–	–	–
Male	805 (40.37)	–	–	–	–
Racial/Ethnic background					
White	1186 (61.01)	7.81	1.80	5.05	13.89
African American	108 (5.56)	7.04	1.36	5.05	12.68
Hispanic	540 (27.78)	8.62	2.22	5.05	13.89
Other or Multiracial	110 (5.66)	7.53	1.69	5.05	12.68

– not applicable. The mean, standard deviation, minimum, and maximum values for each racial/ethnic group represent the walkability values.

### Regression results between CMDs and walkability

Table 2 presents the coefficients, odds ratios (ORs), and 95% confidence intervals (CIs) from the multilevel linear regression and multilevel logistic regression analyses. We found that walkability is significantly and negatively associated with BMI (β = − 0.28, CI − 0.45 to − 0.10). The magnitude of the result shows that when walkability increases by one unit, an individual’s BMI decreases by 0.28 kg/m^2^ on average in our sample. Walkability is also significantly and negatively associated with diabetes (OR 0.93, CI 0.86–0.99). This result indicates that higher walkability is associated with reduced odds of diabetes, with each unit increase in walkability reducing the odds of having diabetes by 7%. We also found that walkability is negatively associated with high blood pressure (OR 0.97, CI 0.91–1.03), heart disease (OR 0.93, CI 0.85–1.02), and CVDs (OR 0.94, CI 0.87–1.02), and positively associated with aerobic physical activity (OR 1.04, CI 0.98–1.11). However, these results are not statistically significant.

**Table 2:** Association between cardiometabolic diseases measures, walkability, and other factors.

Variables	BMI^a^	High blood pressure^b^	Diabetes^c^	Heart disease^d^	CVDs^e^	Physical activity^f^
	Coef. (95% CI)	OR (95% CI)				
Walkability	**− 0.28** * ** (− 0.45 to − 0.10)**	0.97 (0.91–1.03)	**0.93** * ** (0.86–0.99)**	0.93 (0.85–1.02)	0.94 (0.87–1.02)	1.04 (0.98–1.11)
Age	− 0.02 (− 0.04 to 0.00)	**1.05** * ** (1.05–1.06)**	**1.04** * ** (1.03–1.05)**	**1.05** * ** (1.04–1.06)**	**1.05** * ** (1.04–1.06)**	1.00 (0.99–1.01)
Family history of diabetes	–	–	**3.28** * ** (2.44–4.41)**	–	–	–
Sex						
Female	Ref.	Ref.	Ref.	Ref.	Ref.	Ref.
Male	0.17 (− 0.47 to 0.81)	**1.26** * ** (1.03–1.54)**	1.21 (0.92–1.59)	**1.97** * ** (1.43–2.71)**	**1.44** * ** (1.09–1.91)**	**1.44** * ** (1.18–1.75)**
Racial/ethnic groups						
White	Ref.	Ref.	Ref.	Ref.	Ref.	Ref.
African American	0.92 (− 0.49 to 2.33)	**2.93** * ** (1.82–4.71)**	**3.82** * ** (2.29–6.39)**	1.01 (0.50–2.03)	1.21 (0.68–2.16)	**0.57** * ** (0.36–0.90)**
Hispanic	**1.94** * ** (1.17–2.72)**	0.89 (0.69–1.15)	**2.40** * ** (1.74–3.32)**	0.72 (0.46–1.12)	0.77 (0.53–1.12)	**0.75** * ** (0.59–0.96)**
Other or Multiracial	− 0.84 (− 2.24 to 0.56)	1.32 (0.85–2.05)	1.74 (0.99–3.04)	0.68 (0.32–1.46)	0.88 (0.47–1.64)	0.75 (0.49–1.15)

Significant values are in [bold].

– not applicable, *Coef.* coefficient, *Ref.* reference group, *OR* odds ratio, *95% CI* 95% confidence interval.

*Indicates statistically significant associations (*p* < 0.05).

^a^BMI is the dependent variable.

^b^High blood pressure is the dependent variable.

^c^Diabetes is the dependent variable.

^d^Heart disease is the dependent variable.

^e^CVDs is the dependent variable.

^f^Physical activity is the dependent variable. All models include the independent variables of walkability, age, sex, and racial/ethnic groups. Family history of diabetes was included only in the diabetes model.

### Relationship between walkability and racial/ethnic groups

**Fig. 1:**
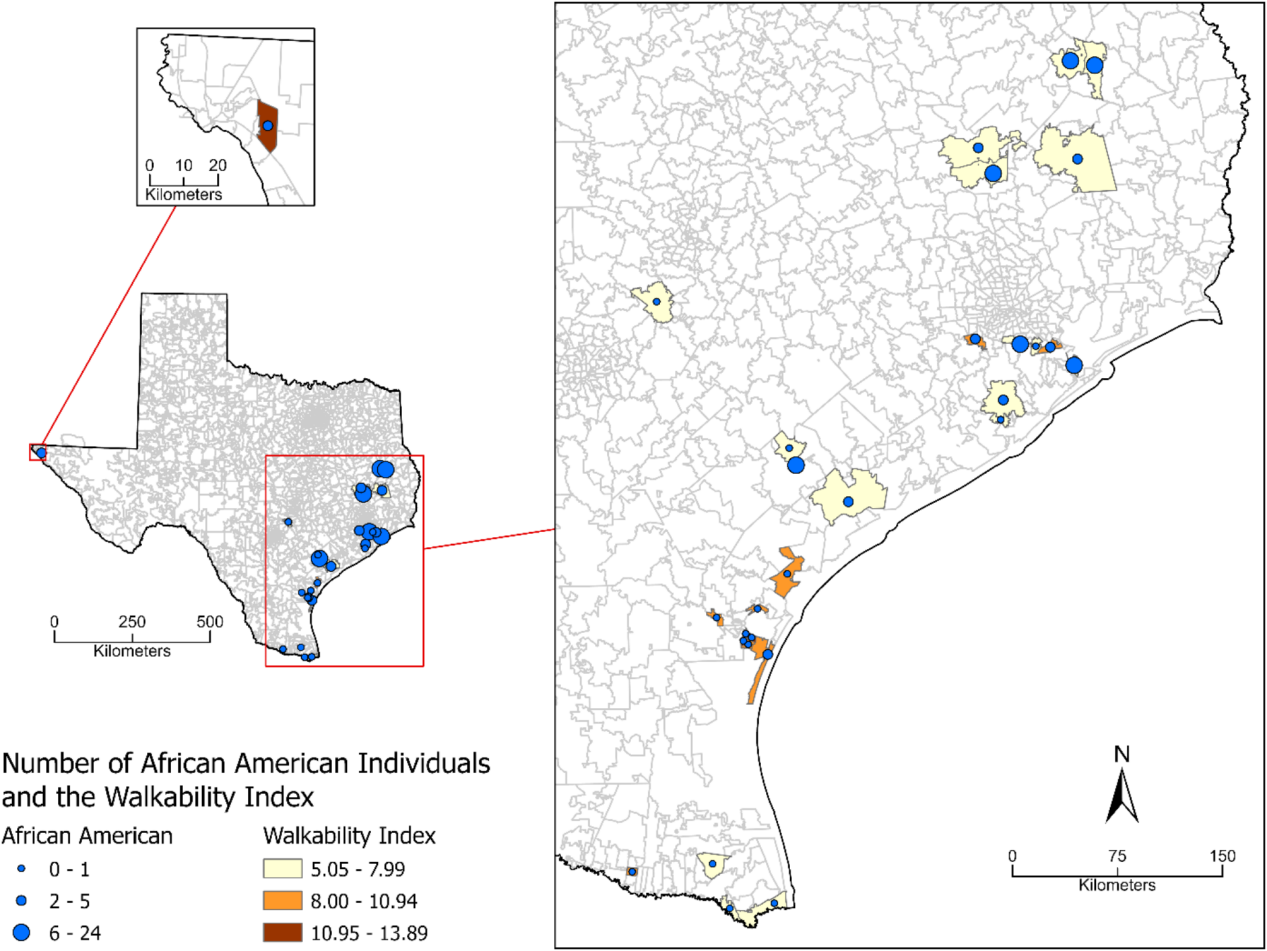
Spatial distribution of the number of African American individuals and the walkability index at the zip code level. Areas with lighter yellow color represent lower walkability, while areas with larger blue dots represent a greater number of African American individuals. Map created using ArcGIS Pro (version 3.0.0, https://www.esri.com/en-us/arcgis/products/arcgis-pro/overview). State and zip code boundary data were collected from the U.S. Census Bureau, Geography Division (https://www.census.gov/cgi-bin/geo/shapefiles/index.php).

The regression results show that, compared to the White participants, the African American individuals (β = − 0.77, CI − 1.15 to − 0.40) had a significant negative association with walkability, while the Hispanic individuals (β = 0.81, CI 0.61–1.00) had a significant positive association with walkability (Table 3). This is consistent with the finding that the African American group had the lowest average walkability, while the Hispanic group had the highest average walkability (Table 1). The Other or Multiracial individuals had a negative association with walkability, but it was not significant. A pairwise correlation results also indicated that African Americans significantly and negatively correlated with walkability (r = − 0.12, *p* < 0.001). Figure 1 shows the spatial distribution of the walkability index and the number of African American individuals in each zip code. The map indicates that areas with lower walkability (light yellow areas) also tend to have more African American populations (larger blue dots). Those results indicate that African Americans live in communities with lower walkability scores.

**Table 3:** Association between walkability and racial/ethnic groups.

Variables	White	African American	Hispanic	Other or Multiracial
Walkability	Coef. (95% CI)			
	Ref.	− 0.77 (− 1.15 to − 0.40)*	0.81 (0.61–1.00)*	− 0.29 (− 0.66 to 0.09)

Significant values are in [bold].

*Coef.* coefficient, *Ref.* reference group, *95% CI* 95% confidence interval.

*Indicates statistically significant associations (*p* < 0.05).

## Discussion and conclusion

In this study, we tested two hypotheses. The first hypothesis is that walkability is negatively associated with CMD outcomes. Our findings support this hypothesis. We found that walkability was negatively and significantly associated with two CMD measures, including BMI and diabetes, suggesting that higher neighborhood walkability is associated with lower diabetes incidence and residents’ BMI. The second hypothesis is that African Americans live in communities with lower walkability scores. We found that African Americans live in communities with significantly lower average walkability than other racial/ethnic groups. This finding supports our second hypothesis. Urban planning strategies, such as promoting more walkable neighborhoods, especially for disadvantaged communities with a high density of African American residents and low walkability scores, can help encourage physical activity while reducing the risk of developing diabetes and high BMI. For individuals already diagnosed with diabetes, increased walking facilitated by more walkable environments can help manage the condition better, potentially reducing the need for medication and lowering the risk of complications. By reducing the risk of developing diabetes and high BMI, walkable neighborhoods can contribute to significant healthcare cost savings, including reduced medical expenses for treatments, hospitalizations, and long-term care. Public health interventions on valuable populations, such as African American and Hispanic, as well as older people, male, and population with family history of diabetes, can help efficiently lower the risk of corresponding CMDs for people at higher risk, and reduce the burden of CMDs incidence and mortality in Texas.

Consistent with our study, neighborhood walkability has been found to benefit certain CMD outcomes, including BMI and diabetes. Previous studies have employed various methods to assess neighborhood walkability and analyze its association with different CMDs measures. For example, walkability has been measured objectively using neighborhood buffers of 500 m^16^, or 800 m and 1600 m^29^. These studies found a significant negative association between a higher neighborhood walkability index and BMI^16^. Individuals living in highly walkable areas had a lower risk of type 2 diabetes mellitus within the 800 buffer^29^. Additionally, z-scores of street connectivity, population density, and land use mix were summed to create a neighborhood active-living environment index or neighborhood walkability score^30,31^. Individuals living in the most active-living-friendly neighborhoods had a BMI that was 0.79 kg/m^2^ lower^30^. Adults in neighborhoods with higher walkability spent more time engaged in physical activity and were less likely to have diabetes^31^. Residents with higher walkability in the “Walker’s Paradise” and “Very Walkable” range had lower odds of being overweight or obese (BMI ≥ 25.0 kg/m^2^)^22^. Neighborhood walkability was also significantly associated with decreased BMI^18^. Another study found that a lower risk of type 2 diabetes was associated with greater cumulative exposure to physical activity resources, such as walking environments and commercial recreational establishments^32^. This indicates that a more walkable neighborhood environment can help reduce the risk of diabetes.

While walkability was significantly associated with BMI and diabetes, its impact on high blood pressure, heart disease, or CVD outcomes was not evident in our study. Previous studies have found that higher walkability was associated with lower systolic and diastolic blood pressure^15,16,18,21,33^, decreased resting heart rate and lower heart disease prevalence^18,34^, and lower predicted 10-year cardiovascular risk^31^. The lack of significant results in our study may be due to the absence of longitudinal data over prolonged periods or the presence of other variables, such as neighborhood violence or insecurity. Future research should repeat the measures with a longitudinal research approach when such data are available and explore additional variables that might interact with walkability to influence these health outcomes.

The results of the control variables in this study align with previous research. Studies have found a positive correlation between older age, being male, and certain CMDs indicators, such as high blood pressure^35,36^, type 2 diabetes^17,29^, heart disease^36,37^, and CVDs^36,38^. Type 2 diabetes is more common in individuals with a family history of the disease^32^. Males were more likely to meet the aerobic physical activity guidelines than females^39^. African American and Hispanic individuals are more vulnerable to type 2 diabetes^32^, and high blood pressure tends to be most prevalent among African Americans^35^. Physical activity can reduce the risk of several CMD indicators^9^, supporting our finding that African American and Hispanic individuals had lower levels of physical activity but a higher risk of diabetes, high BMI, or high blood pressure.

Built environment resources and safety concerns are crucial factors influencing whether residents feel comfortable walking in their neighborhoods^40^. These factors may contribute to the lower walkability observed among African American communities in our study. Economic disparities can lead to differences in built environment resources and neighborhood investment, affecting infrastructure quality, safety, and amenities such as sidewalks, street lighting, and parks, which enhance walkability. Some Black households live in neighborhoods with lower incomes^41^, which may impede their economic mobility and result in fewer built environment resources and more safety concerns, leading to reduced walkability. Additionally, ongoing socioeconomic inequalities contribute to disparities in health outcomes. Studies^42^ show that Black individuals have lower homeownership rates compared to White individuals, and these disparities are associated with poorer health outcomes^43^. Predominantly White neighborhoods tend to have more built environment resources associated with good health compared to neighborhoods with predominantly Black residents^44^. For example, neighborhood walkability, urban development, and green space are linked to improved physical and mental health^45^. However, predominantly Black neighborhoods often have less green space, more dilapidated buildings, more single-lane roads, and higher rates of diabetes and other adverse health outcomes compared to predominantly White neighborhoods^44^. Overall, disparities in socioeconomic status and neighborhood built environment resources not only lead to reduced walkability but also affect the overall health outcomes in African American communities.

Our study reaffirmed the association between walkability, BMI, and diabetes. A key contribution of our study is the inclusion of family history of diabetes, racial/ethnic background, and a diverse range of CMD measures in the analysis, as well as the use of a more comprehensive, and infrastructure-focused neighborhood walkability assessment. This helps bridge some gaps and contributes to the existing body of knowledge. Additionally, the results highlighted vulnerable population groups in specific CMDs, particularly the African American population, who live in communities with lower walkability scores and have a higher risk of high blood pressure and diabetes. Our findings offer critical insights for urban planners and healthcare providers to inform the development of targeted strategies and interventions aimed at mitigating the burden of CMDs in Texas.

A limitation of our study is the insufficient information on the physical activity index. Instead of having a continuous value, our variable is in binary format, indicating whether aerobic recommendations were met (coded as 1) or not (coded as 0). Previous studies found that higher neighborhood walkability can promote physical activity, with a higher prevalence of moderate or high levels of physical activity^16^ and spending more time engaged in physical activity^31^. The binary format of physical activity could be a confounding variable, leading to the insignificant association results between walkability and physical activity. Future studies using more granular measures of physical activity are recommended to analyze its association with walkability more comprehensively. Another limitation is the cross-sectional nature of the data, which prevents establishing causality. Our data was collected in a single year. This type of data can show associations between CMD measures and walkability but cannot establish causal relationships. Future research should consider a longitudinal approach with multi-year data to better understand causal relationships and confirm our findings.

## Conclusion

Overall, our findings indicate that higher neighborhood walkability has a significantly negative association with diabetes incidence and residents’ BMI. By fostering environments that encourage walking, we can make meaningful strides in preventing and managing diabetes, reducing BMI, improving public health, and lowering healthcare costs. The diversity of the participants, encompassing a wide age range, both sexes, and multiple racial/ethnic groups, enhances the representativeness of the results. However, the association between walkability and CMD outcomes across different states in the United States has not yet been explored. We suggest using the National Walkability Index to represent neighborhood walkability and confirm the results in different states when individual-level health records are available, as it uses nationally standardized data, making it valuable for consistent regional comparisons. Additionally, it is crucial to account for several years of data to capture trends and changes over time, further enhancing the representativeness of the findings.

## Data Availability

The National Walkability Index data analyzed during the current study are publicly available from the Environmental Protection Agency (EPA) at https://www.epa.gov/smartgrowth/smart-location-mapping. The cardiometabolic diseases data analyzed during the current study are not publicly available due to the confidentiality agreement required by Texas Behavioral Risk Factor Surveillance System (BRFSS), prohibiting the data user from “distribut(ing) the Texas BRFSS PUDFs to any other partners, organizations, foundations, institutions, agencies or programs.” Researchers interested in the cardiometabolic diseases data supporting the findings of this study can request access directly from the BRFSS at https://healthdata.dshs.texas.gov/dashboard/surveys-and-profiles/behavioral-risk-factor-surveillance-system.
